# COMMUNITY CONTEXT OF OLDER ADULT CARE: A CASE STUDY DURING THE COVID-19 PANDEMIC

**DOI:** 10.1093/geroni/igac059.093

**Published:** 2022-12-20

**Authors:** Lisa Skemp

**Affiliations:** Loyola University, Chicago, Chicago, Illinois, United States

## Abstract

**Introduction:**

Expectations for older adults (OA) to live in the community and prevent costly long-term care assume OAs’ informal network members are available, able, and willing to fill this need. Yet, little is known about the processes whereby OAs construct care networks, especially during COVID-19.

**Methods:**

A longitudinal case study of one OA male who participated in the ethnographic community Older Adult Care study in one urban Chicago neighborhood is described. The OA male described his network on three occasions: 2/2018, 1/2021 and 9/2021. The care networks were described by size, density, and transitivity. Data analysis was performed using the R programming language. Adjacency networks were constructed using the network package, then visualized using the sna package.

**Results:**

The OA’s network went from 23 members pre-pandemic in time one to 13 in time two and 8 members in time three. As network size contracted, the network density increased from 25% in time two to 46% at time three, indicating a more interconnected network. Clustering varied over time and was at its lowest in time 2 (27%) and increased by time 3 (67%). Friends and church connections were 72% of his network in time one, whereas 71% of his network were family and neighbors in time three.

**Conclusions:**

Our OA’s large, relatively disconnected social network tightened to fewer, more closely connected members during COVID-19 months. Contextual variables (environment, health guidelines, fear, pets) influenced the networks. It is essential to understand OA care networks to promote healthy aging in community.

